# Comparable Stability of Hoogsteen and Watson–Crick Base Pairs in Ionic Liquid Choline Dihydrogen Phosphate

**DOI:** 10.1038/srep03593

**Published:** 2014-01-08

**Authors:** Hisae Tateishi-Karimata, Miki Nakano, Naoki Sugimoto

**Affiliations:** 1 Frontier Institute for Biomolecular Engineering Research (FIBER), Konan University, 7-1-20 Minatojima-minamimachi, Chuo-ku, Kobe, 650-0047, Japan; 2 Faculty of Frontiers of Innovative Research in Science and Technology (FIRST), Konan University, 7-1-20 Minatojima-minamimachi, Chuo-ku, Kobe, 650-0047, Japan

## Abstract

The instability of Hoogsteen base pairs relative to Watson–Crick base pairs has limited biological applications of triplex-forming oligonucleotides. Hydrated ionic liquids (ILs) provide favourable environments for a wide range of chemical reactions and are known to impact the stabilities of Watson–Crick base pairs. We found that DNA triplex formation was significantly stabilized in hydrated choline dihydrogen phosphate as compared with an aqueous buffer at neutral pH. Interestingly, the stability of Hoogsteen base pairs was found to be comparable with that of Watson–Crick base pairs in the hydrated IL. Molecular dynamics simulations of a DNA triplex in the presence of choline ions revealed that the DNA triplex was stabilized because of the binding of choline ion around the third strand in the grooves. Our finding will facilitate the development of new DNA materials. Our data also indicate that triplex formation may be stabilized inside cells where choline ions and their derivatives are abundant *in vivo*.

In solution at physiological pH, the canonical DNA structure is a B-form duplex consisting of A–T and G–C Watson–Crick base pairs (the en dash indicates Watson–Crick base pair)^1^, and these Watson–Crick base pairs conserve genetic information. DNA has enormous potential in nanobiotechnology and biomedical technology because single strands of DNA can recognize and hybridize with their complementary sequences through highly specific base-pairing interactions^2^,^3^,^4^,^5^,^6^. Nanoarchitectures based on Watson–Crick base pairs are used to create biomaterials for use in separations, controlled drug delivery, diagnostics and as interfacial materials between biological and inorganic matter^7^,^8^,^9^,^10^.

Nucleic acids can also form Hoogsteen base pairs^11^. For example, in a triple helix, a third strand, called triplex-forming oligonucleotide, binds with sequence specificity to A*T and G*C Hoogsteen base pairs (the asterisk indicates the Hoogsteen base pair) in the major groove of a Watson–Crick base-paired DNA duplex^11^,^12^,^13^,^14^. Although previous studies have hypothesized that Hoogsteen base pairs and triplex structures play an important role in transcription, replication and other cellular processes, Hoogsteen base pairs and triplexes are not considered common structures^8^,^15^. Some proteins have evolved to recognize only one type of base pair, and they use intermolecular interactions to shift the equilibrium between Watson–Crick and Hoogsteen geometries^16^,^17^,^18^. The development of DNA materials and diagnostic applications based on the formation of Hoogsteen base pairs has been challenging as these base pairs are stable only in certain sequence motifs and at low pH^19^,^20^. For example, in polypurine tracts, found in human immunodeficiency virus-1 (HIV-1) proviral and triplet repeat diseases, cytosines in the third strand must be protonated at N3 (p*K*_a_ = 4.5) to form C–G*C^+^ base triplets^8^,^14^. As the formation of Hoogsteen base pairs of mixed G and A sequences is not stable at neutral pH^14^, biological applications have been limited.

Nucleic acids in an aqueous solution are not sufficiently stable for practical use in many applications as these molecules spontaneously degrade^21^,^22^ or are degraded by a contaminating nuclease. Consequently, chemical instability is a bottleneck in the development of nanotechnology based on nucleic acids^23^. Furthermore, aqueous solutions are impractical in small-volume solution technologies because small volumes of water immediately vaporize under open air conditions or at high temperatures^21^. Some remarkable features of ILs make these liquids attractive alternatives to water for various applications such as in electrochemistry, separation science, chemical synthesis and materials science^24^,^25^,^26^,^27^. ILs improve solute properties such as solubility, stability and bioavailability. For example, choline dihydrogen phosphate (or choline dhp), a representative IL, dissolved in a small amount of water (approximately 20 wt%) ensures the long-term stability of biomolecules such as DNA and has negligible vapour pressure^26^,^28^,^29^. We recently proved that A–T base pairs are more stable than G–C base pairs in a hydrated choline dhp solution because of specific stabilizing interactions between choline and bases in both grooves^30^,^31^. Recently, a triplex consisting of T–A*T base triplets is stabilized in a deep eutectic solvent containing a choline ion similar to an IL has been reported^32^. Further studies have suggested that molecules that bind to the grooves of A–T-rich duplexes could bind to the major and minor grooves in a triplex, thus stabilizing the triplex structure^33^,^34^,^35^,^36^.

We investigated the formation of DNA triplexes in the hydrated IL of choline dhp using thermodynamic analyses and molecular dynamics (MD) simulations. We found that the hydrated IL stabilized the formation of Hoogsteen base pairs via specific interactions between choline ion and the triplex. Use of ILs as stabilizers of Hoogsteen base pairs will enable the formation of useful nucleic-acid devices. Our findings should also enable the development of diagnostic tools based on triplex formation. The results are also biologically relevant: The fact that Hoogsteen base pairs are stable at neutral pH in choline dhp suggests that these base pairs may be formed in cells because choline ions and their derivatives are abundant *in vivo*.

## Results

### Choline dhp influences the stability of Watson–Crick and Hoogsteen base pairs distinctively

**Figure 1:**
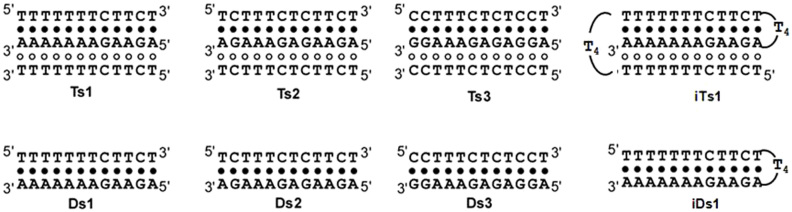
Sequences and schematic structures of triplexes (Ts1, Ts2, Ts3 and iTs1) and duplexes (Ds1, Ds2, Ds3 and iDs1). Filled and open circles indicate Watson–Crick and Hoogsteen base pairs, respectively.

We designed and synthesized oligonucleotides to form three intermolecular DNA triplexes (Ts1, Ts2 and Ts3) with different G*C base pair content (Figure 1). iTs1, which has the same sequence as Ts1 except for the existence of the loop region was also synthesized; this DNA can form an intramolecular triplex (Figure 1). Three intermolecular double-stranded DNAs (Ds1, Ds2 and Ds3) and one intramolecular double-stranded DNA (iDs1) with the same sequences of Watson–Crick base pairs as Ts1, Ts2, Ts3 and iTs1 were also prepared (Figure 1).

**Figure 2:**
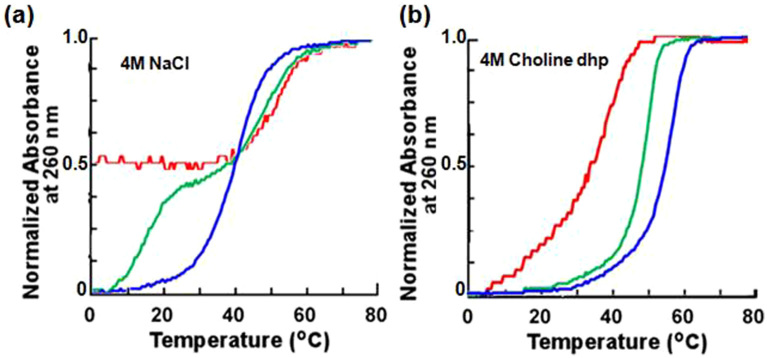
Thermal stability of DNA triplexes. Normalized UV melting curves at 260 nm for Ts1 (blue), Ts2 (green) and Ts3 (red) in a (a) 4 M NaCl solution and (b) 4 M choline dhp solution. Solutions also contained 50 mM Tris (pH 7.0) and 1 mM Na_2_EDTA. Total DNA strand concentration was 30 μM.

We investigated the thermal stability of the triplexes in 4 M (80 wt%) choline dhp because solutions of choline dhp with this concentration have decreased water activity, lowered dielectric constants and altered ion networks relative to an aqueous buffer^37^. Figure 2 shows normalized UV melting curves at 260 nm for 30 μM triplexes. We also measured UV melting curves at 295 nm (Figure S1) because the dissociation of Hoogsteen base pairs can be specifically monitored at this wavelength^38^. A melting curve for triplexes has two sigmoidal melting transitions: one for the melting of the duplex and the other for the dissociation of the triplex strand. If a transition at 260 nm occurs with the same midpoint (melting temperature, *T*_m_) as the melting transition observed at 295 nm, this transition corresponds to the dissociation of Hoogsteen base pairs (*T*_m-H_). A transition observed only at 260 nm corresponds to the dissociation of Watson–Crick base pairs (*T*_m-W_). If the UV melting curve at 260 and 295 nm has single transitions with very similar *T*_m_s, the Hoogsteen and Watson–Crick base pairs dissociate at the same temperature (*T*_m-H&W_). The melting temperatures of the analysed duplexes and triplexes are shown in Tables 1 and S1.

The melting curves of Ts1 in 4 M NaCl at 260 nm and 295 nm showed a single sigmoidal melting transition with an approximately equal *T*_m_s (Figure 2, Table 1 and Figure S1), suggesting that Watson–Crick and Hoogsteen base pairs dissociated at the same time. The melting curves for Ts2 in 4 M NaCl at 260 nm showed two sigmoidal melting transitions. The lower-temperature transition corresponded to the dissociation of Hoogsteen base pairs because the *T*_m_ value was similar to that of the transition at 295 nm, whereas the higher-temperature transition corresponded to the dissociation of Watson–Crick base pairs. Ts3 in a 4 M NaCl solution showed one sigmoidal melting transition at 260 nm but no transitions at 295 nm, indicating that third-strand binding via Hoogsteen base pairing did not occur in NaCl solution. Moreover, for Ts3 in a 4 M NaCl solution, the *T*_m_ of Ds3 was almost identical to that at 260 nm (Table 1). This also indicated that Ts3, which had the highest G*C base pair content, does not form a triplex^13^. Triplex formation was also confirmed by circular dichroism (Figure S2)^38^.

In contrast, the UV melting curves for Ts1, Ts2 and Ts3 in a 4 M choline dhp solution showed a single sigmoidal melting transition with approximately the same *T*_m_s at 260 nm and 295 nm (Tables 1 and S1). In the hydrated IL, therefore, Ts1, Ts2 and Ts3 formed triplexes even at pH 7.0, and the stability of Hoogsteen base pairs was found to be comparable to that of Watson–Crick base pairs. The extent of differences in stability in NaCl vs. choline dhp depended on the G–C base pair content and probably resulted from specific interactions between DNA choline ions and bases^30^. The *T*_m-H&W_ values for Ts1 and Ts2 in the choline dhp solution were higher than the *T*_m-W_ values for Ds1 and Ds2 (Table 1). Ts3 formed a triplex in a choline dhp solution but not in a NaCl solution. In choline dhp, the stability of Watson–Crick base-paired duplexes depended on the G–C base pair content; however, choline dhp stabilized the formation of Hoogsteen base pairs independent of sequence^30^. The stabilization of Hoogsteen base pairs in choline dhp is significant compared with that observed in previous studies with triplex-forming oligonucleotides with DNA backbone modifications^11^. We also evaluated the melting of Ts1, Ts2 and Ts3 at pH 7.0 in 4 M choline chloride, which is not a hydrated IL. The *T*_m_ values of all triplexes increased in the choline chloride solution relative to the NaCl solution (data not shown), although choline dhp stabilized the triplex structure more significantly than choline chloride. In both cases, the stabilization may be due to an interaction between choline ions and DNA.

To understand how high concentrations of choline dhp altered the stabilities of DNA structures, we measured the thermodynamic parameters for intramolecular triplex iTs1 and duplex iDs1 in 4 M NaCl and choline dhp (Table 2). The Δ*G*°_25_ values (free energy change at 25°C) for the structure formation of iTs1 and iDs1 in 4 M NaCl were −13.1 and −8.8 kcal mol^−1^, respectively, suggesting that the triple-strand binding of iTs is more stable than the double-strand binding of iDs1. The Δ*G*°_25_ values for the structure formation of iTs1 and iDs1 in 4 M choline dhp were −17.5 and −8.9 kcal mol^−1^, respectively. The Δ*G*°_25_ values of iTs1 indicate that the triplex of iTs1 was significantly more stable in choline dhp than in the NaCl solution. As stabilization of the triplex in choline dhp was enthalpically driven, choline ions may increase the stability of triplex because of an interaction between choline ions and DNA atoms in the triplex. Previous studies of triplexes in the presence of spermine^39^, acridine^33^, Hoechst 33258^34^, neomycine^33^ and carbon nanotubes^40^ indicated that small molecules increased triplex stability through enthalpic contributions. For example, triplex stabilization by neomycine was up to 5 kcal mol^−1^ (Δ*G*°_25_). Here, we demonstrated that choline dhp stabilized the triplex more effectively than the small molecules evaluated previously^39^,^40^.

### Interaction between choline ions and triplex evaluated by molecular dynamic simulations

To understand how choline ions stabilize triplex structures, we performed 20 ns MD simulations of DNA triplexes, Ts1, Ts2 and Ts3, with choline ions (see Supporting Information). To maintain the triplex structures during simulation, the N3 in cytosine in the third strand was protonated. For each triplex, we counted the number of choline and sodium ions within 3.5 Å of the DNA strand. A large number of choline ions bound to the triplexes: 27.8 ions bound to Ts1, 30.6 ions bound to Ts2 and 26.9 ions bound to Ts3 (Table 3). Fewer sodium ions bound to Ts1, Ts2 and Ts3 (9.8, 7.9 and 9.5, respectively; Table 3). Despite differences in the sequences of these triplexes, the number of bound choline and sodium ions was not significantly different.

**Figure 3:**
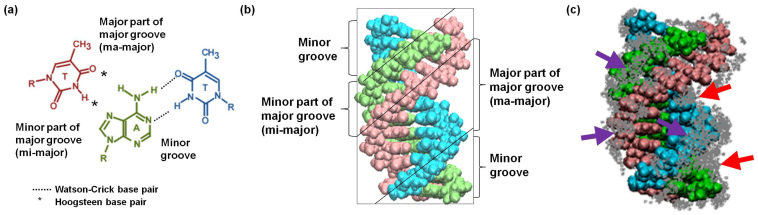
Triplex structure and interaction between choline ions and triplexes. (a) The bases of the triplet of T–A*T are indicated by light blue, light green and pink. (b) The structure of Ts1 is depicted by the van der Waals model. First (5′-TTTTTTTCTTCT-3′), second (5′-AGAAGAAAAAAA-3′) and third (5′-TCTTCTTTTTTT-3′) strands are indicated by light blue, light green and pink, respectively. (c) Binding sites of choline ions around Ts1. Grey dots represent the appearance frequency of choline ions. Red and purple arrows indicate the highlighted binding site.

For this analysis, the grooves of a triplex are defined as the major part of a major groove (ma-major groove), minor part of a major groove (mi-major groove) and minor groove (Figures 3a and 3b)^12^. We measured the widths of minor, mi-major and ma-major grooves (distances between the phosphate groups in the first and second strands, second and third strands, or first and third strands, respectively) in triplexes because we previously found that the binding of choline ions strongly depended on the width of the minor groove of the duplex^31^. There were no significant differences in the widths of minor, mi-major and ma-major grooves in the presence of choline ions (Ts1, T2 and Ts3 had minor, mi-major ma-major groove widths of 12.7 to 13.0 Å, 8.5 to 8.8 Å and 14.0 to 15.3 Å, respectively; Table S2). These results suggest that choline ion affinity did not depend on a triplex sequence. Although choline ions preferentially bind to A–T base pairs than to G–C base pairs in a DNA duplex, as shown in our previous report^31^, in these simulations with triplexes, choline ion binding was not sequence-specific.

To evaluate the binding mode of choline ions to triplexes in greater detail, we analysed the binding position of choline and sodium ions around triplexes from 25000 snapshots taken from trajectories at 15–20 ns intervals of the MD simulations. The total number of choline and sodium ions around the triplexes in the 25000 snapshots is represented by grey dots in Figures 3c and S3. Since the binding modes to each triplex were similar, we focused on Ts1. The high-frequency binding sites of choline ions are highlighted by arrows in Figure 3c. Choline ions bind around each strand, particularly the third strand, suggesting that choline ions bind to phosphates (Figure 3c, purple arrows). Interestingly, the choline ions also bind deep inside both the minor and ma-major grooves (Figure 3c, red arrows). In contrast, sodium ions are primarily bound to phosphate groups. In addition, as compared to choline ions, significantly fewer sodium ions are associated with the minor and ma-major grooves (Figure S3). The stabilization of the triplex by the choline ions is discussed in detail in the Discussion section.

To quantitatively investigate how ion binding affects the third-strand binding to the duplex, we calculated the energy changes of third-strand binding to the duplex with choline or sodium ions (Δ*E*) using the molecular mechanics-generalized Born surface area (MM-GBSA) module in AMBER 12 (see Supporting Information). The strength of third-strand binding depends on the protonation of cytosine. It is difficult to experimentally investigate the ion effect on third-strand binding because protonation efficiency depends on solution conditions. However, the Δ*E* values in our simulation system can be used to estimate how ions affect third-strand binding by ignoring the protonation of cytosine. The Δ*E* values for Ts1, Ts2 and Ts3 with choline ions were −115, −115 and −129 kcal mol^−1^ and those with sodium ions were −61.3, −83.5 and −84.7 kcal mol^−1^, respectively (Table 3). Thus, MM-GBSA analysis showed that the interaction between the third strand and choline ions enhanced triplex stability more than that between the third strand and sodium ions in a way that was almost independent of DNA sequence.

## Discussion

### Choline ions stabilize triplex structures

We previously reported that choline ions stabilize A–T base pairs in a DNA duplex but destabilize G–C base pairs. MD simulations indicate that choline ions preferentially bind to A–T base pairs in the minor groove and enhance the stability of A–T base pairs^31^. In contrast, the choline ions preferentially bind to G in single strands as well as G–C base pairs from the major groove side, thus destabilizing the hydrogen bonds of the G–C base pairs^30^,^31^. Our present results show that choline dhp stabilizes triplex formation even for triplexes containing G–C base pairs. The major groove side of the G–C base pairs interacts with the third strand; thus, the choline ions cannot attack the hydrogen bonds in the G–C base pairs.

**Figure 4:**
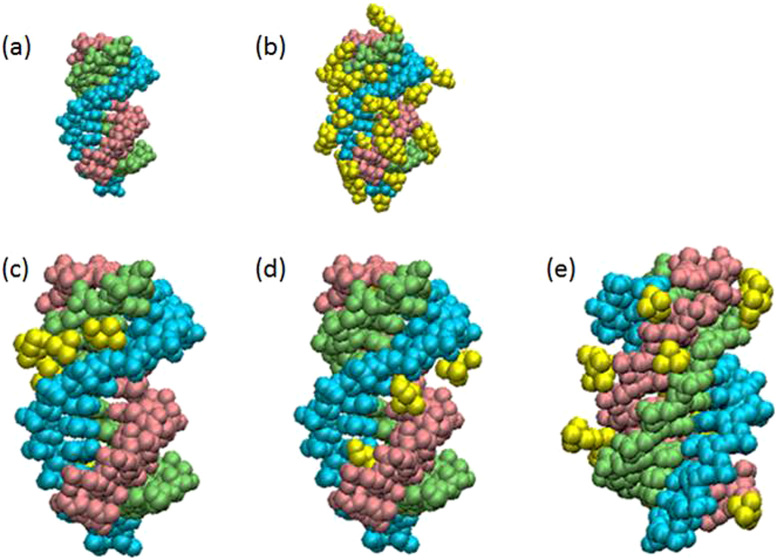
Estimation of Binding of Choline ions to the triplex by MD simulations. A snapshot of Ts1 after 20 ns MD simulation in (a) the absence and (b) presence of choline ions. First (5′-TTTTTTTCTTCT-3′), second (5′-AGAAGAAAAAAA-3′) and third (5′-TCTTCTTTTTTT-3′) strands in Ts1 are indicated by light blue, light green and pink, respectively. Ts1 and choline ions (yellow) are depicted as van der Waals models. The choline ions bound to minor and ma-major grooves in Ts1 are highlighted in (c) and (d), repectively. The choline ions surrounding the third strand in Ts1 are highlighted in (e).

To microscopically analyse choline ion binding to Ts1, we selected and analysed a snapshot of Ts1 after 20 ns and the final snapshot in MD simulations (Figure 3c). Snapshots of Ts1 after 20 ns MD simulations in the absence and presence of choline ions are shown in Figure 4a and 4b, respectively. Choline ions are bound with high frequency to three types of binding sites in Ts1. The choline ions are buried inside the minor groove (Figure 4c) and the ma-major groove (Figure 4d) and surround the third strand (Figure 4e). It was reported that alkylammonium ions such as trimethyl ammonium ions bind to A–T base pairs in the minor groove of a DNA duplex to stabilize the duplex^41^,^42^. As observed previously, both the minor and mi-major grooves of a triplex are quite rigid because of the existence of highly structured water molecules in both grooves^39^. In contrast, the ma-major groove is quite flexible. The binding of small molecules in the ma-major groove probably enhances triplex stability independent of sequence^39^,^40^. Similarly, the choline ions bridge the first and second strands or the first and third strands to strengthen and stabilize triplex formation.

The choline ions in the mi-major groove surround the phosphates of the third strand (Figure 4e). The mi-major groove appears to be too narrow for a choline ion to be buried inside. Generally, the cations primarily bind to nucleotide phosphates and stabilize the ordered DNA structures by reducing the repulsive forces between the phosphate groups^43^,^44^. Cations should accumulate in the mi-major groove because the distances between phosphates across the mi-major groove are shorter than those across the other grooves. The sodium ions rapidly exchange with ions in the bulk solution^44^. As the hydroxyl group in choline ion is strongly polarized, the hydroxyl group forms hydrogen bonds and does not exchange rapidly^31^,^45^. A typical binding mode of a choline ion to the third strand in Ts1 is shown in Figure S4. The hydroxyl group in the choline ion forms a hydrogen bond with the backbone of the third strand in Ts1. The choline ions are located near the third strand and are significantly longer in comparison with sodium ions. MM-GBSA analysis showed that the binding energy of the third strand was enhanced more in the presence of choline ions than sodium ions. In an aqueous solution at neutral pH, triplexes with G*C Hoogsteen base pairs are unstable without the protonation of cytosines. However, in a choline dhp solution, choline ions surround the third strand and strengthen its binding to the duplex, thereby stabilizing the G*C base pairs at neutral pH. Since choline ions have a methyl group, choline ion binding might result in a local hydrophobic environment around the third strand. This environment might prompt the protonation of cytosines. Thus, binding of choline ions to the third strand is stabilized independent of sequence.

### Utility of hydrated ILs for DNA nanotechology and diagnostic devices

In this study, we showed that the hydrated IL of choline dhp resulted in stable Hoogsteen base pairing. Because of its unique properties, choline dhp is an alternative to an aqueous solution in DNA nanotechnology. The self-assembly properties of DNA have been exploited for the preparation of nanosized objects and arrays, which offer the potential to act as scaffolds for the spatial positioning of molecular components with nanometer precision^46^. Thus, specific modifications of nanoarchitectures based on DNA are necessary for the development of new functional materials and devices. Although chemical modifications to DNA may be useful, the methods are complicated^47^,^48^. Triplex formation stabilized by ILs can be exploited to spontaneously recognize specific sequences within DNA nanoarchitectures^49^.

ILs have several properties that will enhance the functions of DNA nanodevices. Natural DNAs are not chemically stable in solution at ambient temperatures for long periods. Thus, DNA devices are not generally reuseable for multiple cycles. MacFarlane et al. reported that DNA has long-term stability in choline dhp in the absence of nuclease^29^. We also found that the nuclease degradation of DNA was significantly inhibited in a 4 M choline dhp solution (unpublished result). It has also been reported that high salt concentrations reduced the activity of enzymes that act on DNA, such as DNA polymerase, because the enzyme structure was destabilized by the high salt concentrations^50^. Nuclease stability may be similarly reduced in choline dhp. Thus, choline dhp could be useful as both a triplex stabilizer and nuclease inhibitor. Another important property of ILs is their low vapour pressure, making them better solvents than water for low-volume devices.

Systems for sensing particular DNA sequences are important in medicine and nanobiosensing^5^,^6^,^51^,^52^. Traditional methods for sensing a DNA sequence, such as DNA microarrays, Southern blots and *in situ* hybridization, are based on the formation of Watson–Crick base pairs and require the generation of single-stranded DNA prior to analysis^2^. To simplify target detection, many approaches have been developed in which double-stranded targets are detected directly^8^,^9^. These systems use intercalating dyes and groove-binding ligands; however, such agents lack sequence specificity; moreover, they are prone to false-positive detection^53^,^54^. A triplex is a promising recognition motif for the sequence-specific sensing of double-stranded DNA targets^8^,^9^. Electrochemical sensors for the detection of a target duplex such as HIV-1 PCR amplicons that employ triplex-forming oligonucleotides have been reported, although a special instrument and synthesis technique were required^8^,^9^. Based on our finding of the significant stabilization of Hoogsteen base pairs with choline dhp, the sequence-specific sensing of double-stranded DNAs without requiring the denaturation of the duplex or complicated instrumentation will be possible.

From a biological viewpoint, our data may be also relevant to the environment in which DNA is found in cells because osmolytes, such as choline ions and glycine betaine, which is an alkylammonium ion derivative, are in abundant supply in cells^55^,^56^, and phosphatidylcholine is present in the nuclear membrane. Probably certain regions of DNA inside the nucleus form triplexes to regulate biological reactions such as transcription. If there is an interaction between osmolytes and DNA or nuclear membranes and DNA, our data suggest that Hoogsteen base pair formation will be stabilized.

## Methods

### Materials

All oligodeoxynucleotides used in this study were of high-performance liquid chromatography grade (Japan Bio Service). Single-strand DNA oligonucleotide concentrations were determined from absorbance at 260 nm at 80°C, and single-strand extinction coefficients were calculated from the mononucleotide and dinucleotide data according to the nearest-neighbour approximation model. The absorbance was measured using a Shimadzu 1700 spectrophotometer connected to a thermoprogrammer. The hydrated IL choline dhp was purchased from Ionic Liquids Technologies Co. Ltd. and used without further purification. between 0.2 and 0.5°C min^−1^ (data not shown). Prior to measurement, the DNA samples were heated to 80°C, cooled to 0°C at a rate of −0.5°C min^−1^ and incubated at 0°C for 30 min.

The melting curve of Ts3 in 4 M NaCl was normalized in the range 0.5–1.0 because the hypochromicity of Ts3 in 4 M NaCl was approximately half of those for Ts1 and Ts2 in 4 M NaCl (Figure 2a).

### Molecular dynamics simulations

For MD simulations, we built a model of DNA triplexes using the nucleotide builder module in Discovery Studio version 3.1. TIP3 water molecules were added at a distance of 20 Å from the DNA triplex using the LEaP module included in AmberTools 12. Two hundred water molecules were randomly replaced with choline or sodium ions (Table S3). To neutralize the simulation system, a corresponding number of water molecules were replaced with chloride ions (Table S3). Figure S5a and S5b show the initial structure for Ts1 containing choline ions and Na^+^, respectively. Figure S5b shows the properties of our simulation systems.

Simulations were conducted with the AMBER 12 software package. The force field of the choline ion was generated using the antechamber module in AmberTools 12 in conjunction with the general AMBER force field. AMBER ff99 force field was applied for DNA during the simulations^55^,^56^. The protocols of structural optimization and MD simulations were as follows. First, the optimization of water molecules and ions was carried out in 1000 steps with a fixed DNA structure. Second, the whole system was minimized in 2500 steps without constraints. Third, the system was heated to 300 K for 20 ps while maintaining weak restraints on the DNA structure without constraints. The constant-pressure and -temperature MD simulation was conducted at 1 atm and 300 K for 10 ns without constraints. Throughout these MD simulations, periodic boundary conditions and the SHAKE algorithm were applied. The simulation time step was set to 2 fs, and the non-bonded cutoff length was set at 10 Å.

For each simulation system, we calculated the accumulated averages of the number of cations within 3.5 Å from DNA atoms, 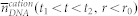, where 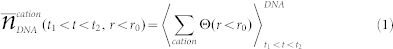
for 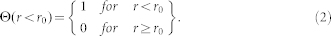
Here *r* is the distance between the target cation and DNA atom, and *r*_0_ is the threshold distance of *r*. We set *r*_0_ to 3.5 Å. Figure S6 shows 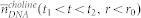 and 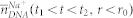 values with a simulation time course for Ts1. For analysis, we used 25000 snapshots from a 15 to 20 ns MD trajectory when the system was in equilibrium.

Using the MM-GBSA module in AMBER 12, we calculated the binding energies between a duplex with Watson–Crick base pairs and a third strand with cations for Ts1, Ts2 and Ts3. 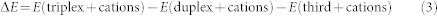
The molecular mechanical energies were determined with the sander program from AMBER 12; they represent internal energies (bond, angle and dihedral) and the van der Waals and electrostatic interactions. The electrostatic contributions to the solvation free energy were calculated by generalized Born methods. Non-polar contributions to the solvation free energy were determined with solvent-accessible surface-area-dependent terms. We selected cations closest to the triplex, i.e. the number required to neutralize the system, and divided these cations for the duplex and third strand (22 cations for duplex, 9 for third strand of Ts1, 7 for third strand of Ts2 and 5 cations for third strand of Ts3).

## Author Contributions

H.T.-K. and N.S. designed the research and wrote the paper. H.T.-K. and technical assistants (see Acknowledgements) performed most of the experiments, while M.N. performed the molecular dynamics simulations of interaction between the triplexes and choline ions. All experiments and discussions were conducted under the supervision of N.S.

## Supplementary Material

Supplementary Information Supporting information

## Figures and Tables

**Table 1:** Melting temperatures of DNA triplexes and duplexes in 4 M NaCl and 4 M choline dhp^a^

	*T*_m_(°C) at 260 nm^b^				
Condition/Sequence^b^	*T_m-H_*	*T_m-W_*	*T_m-H&W_*	*T_m-W_*	
4 M NaCl					
Ts1 (2)^c^			39.4	Ds1 (2)^d^	43.8
Ts2 (4)	14.5	48.1		Ds2 (4)	47.5
Ts3 (6)	n.d.^e^	51.6		Ds3 (6)	51.2
4 M choline dhp					
Ts1 (2)^c^			55.5	Ds1 (2)^d^	51.4
Ts2 (4)			49.3	Ds2 (4)	43.2
Ts3 (6)			37.3	Ds3 (6)	40.2

^a^All experiments were conducted in a buffer containing 50 mM Tris (pH 7.0), 1 mM Na_2_EDTA and 4 M NaCl or 4 M choline dhp.

^b^Total DNA strand concentration of triplexes and duplexes were 30 and 20 μM, respectively.

^c^The number of G*C Hoogsteen base pairs is shown in parentheses.

^d^The number of G–C Watson–Crick base pairs is shown in parentheses.

^e^n.d. indicates that the *T*_m-H_ was too low to be determined.

**Table 2:** Thermodynamic parameters for the formation of DNA triplexes and duplexes measured in 4 M NaCl or 4 M choline dhp^a^

	Δ*H*° (kcal mol^−1^)	*T*Δ*S*° (kcal mol^−1^)	Δ*G*°_25_ (kcal mol^−1^)	*T*_m_^b^ (°C)
4 M NaCl				
iTs1	−80.3 ± 8.7	−67.3 ± 7.7	−13.1 ± 2.5	78.5
iDs1	−57.2 ± 1.1	−48.4 ± 0.9	−8.8 ± 0.7	75.3
4 M Choline dhp				
iTs1	−96.3 ± 8.7	−78.8 ± 7.7	−17.5 ± 2.5	83.9
iDs3	−57.4 ± 1.0	−48.5 ± 0.9	−8.9 ± 0.7	77.3

^a^All experiments were conducted in a buffer containing 50 mM Tris (pH 7.0), 1 mM Na_2_EDTA and 4 M NaCl or 4 M choline dhp. Thermodynamic parameters were evaluated from curve fitting.

^b^Melting temperature was calculated at a strand concentration of 30 μM.

**Table 3:** Number (*N*) of choline and sodium ions within 3.5 Å of Ts1, Ts2 and Ts3 DNA strands, and the energy changes (Δ*E*) of third-strand binding to duplex with choline or sodium ions

	NaCl		Choline dhp	
	*N*	Δ*E* (kcal mol^−1^)	*N*	Δ*E* (kcal mol^−1^)
Ts1	9.8±2.3	−61.3±6.7	27.8±3.5	−115±11
Ts2	7.9±2.2	−83.5±5.8	30.6±3.0	−115±15
Ts3	9.5±2.4	−84.7±6.4	26.9±4.0	−129±14
