# Supplementary material for: Comparable Stability of Hoogsteen and Watson–Crick Base Pairs in Ionic Liquid Choline Dihydrogen Phosphate
Source: Sci Rep. 2014 Jan 8;4:3593. doi: 10.1038/srep03593 (PMC3884231; doi:10.1038/srep03593)
Supplement: Supplementary Information — Supporting information [file srep03593-s1.pdf]

Supporting Information

Comparable Stability of Hoogsteen and Watson–Crick Base Pairs in Ionic Liquid Choline Dihydrogen Phosphate

Hisae Tateishi-Karimata,[1] Miki Nakano,[1] and Naoki Sugimoto[1,2*]

[1]*Frontier Institute for Biomolecular Engineering Research (FIBER) and* [2]*Faculty of Frontiers of Innovative Research in*

Science and Technology (FIRST), Konan University, 7-1-20 Minatojima-minamimachi, Chuo-ku, Kobe 650-0047, Japan

Table S1. Melting temperatures of DNA triplexes at 295 nm in the presence of 4 M NaCl or 4 M choline dhp [a]

Sequence name	T_m (°C) in NaCl	T_m (°C) in choline dhp
Ts1	38.8	56.2
Ts2	15.0	49.0
Ts3	n.d. [b]	39.2

[a] Melting temperatures were determined at strand concentration of 30 μM in a buffer containing 50 mM Tris (pH 7.0), 1 mM Na_2EDTA and 4 M NaCl or 4 M choline dhp.

[b] n.d. indicates that the T_m was too low to be determined.

Table S2. Widths (Å) of minor and mi-major grooves (distance of the phosphates in the first and second strands or first and third strands) in triplexes

**Sequence name**	**NaCl**			**Choline dhp**		
	**minor**	**mi-major**	**mi-major**	**minor**	**mi-major**	**mi-major**
Ts1	11.9 ± 1.0	9.2 ± 1.0	14.4 ± 1.4	13.0 ± 1.1	8.5 ± 1.4	15.3 ± 2.2
Ts2	12.5 ± 1.1	9.0 ± 0.9	14.4 ±1.7	12.7 ± 1.3	8.7 ± 1.4	15.3 ± 2.3
Ts3	12.5 ± 1.1	8.7 ± 0.9	14.2±1.7	13.0 ± 1.1	8.8 ± 0.9	14.0 ± 1.7

Table S3. Names and properties of the models used in the molecular dynamics simulations

**Name(cation)**	**Number of water molecules**	**Number of cations**	**Number of chloride ions**	**Number of atoms**
Ts1(choline$^+$)	6169	200	169	30651
Ts2(choline$^+$)	6091	200	171	30249
Ts3(choline$^+$)	6173	200	173	30577
Ts1(Na^+)	6140	200	169	26445
Ts2(Na^+)	6158	200	171	26517
Ts3(Na^+)	6158	200	173	26517

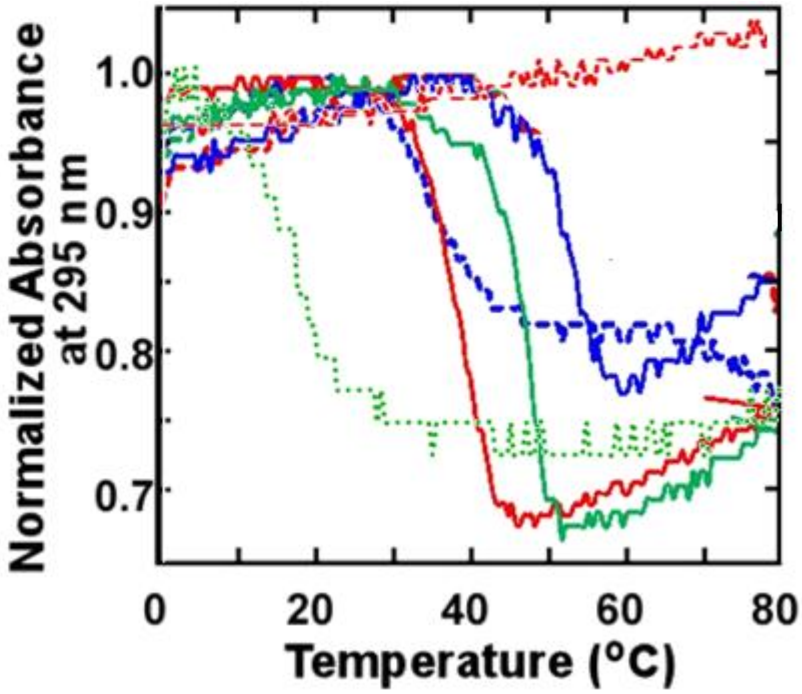

Figure S1. Normalized UV melting curves for Ts1 (blue), Ts2 (green) and Ts3 (red) in NaCl (dashed line) and choline dhp (solid line) solutions at 295 nm. Solutions were 50 mM Tris (pH 7.0), 1 mM Na_2EDTA and 4 M NaCl or 4 M choline dhp. Total DNA strand concentration was 30 μM.

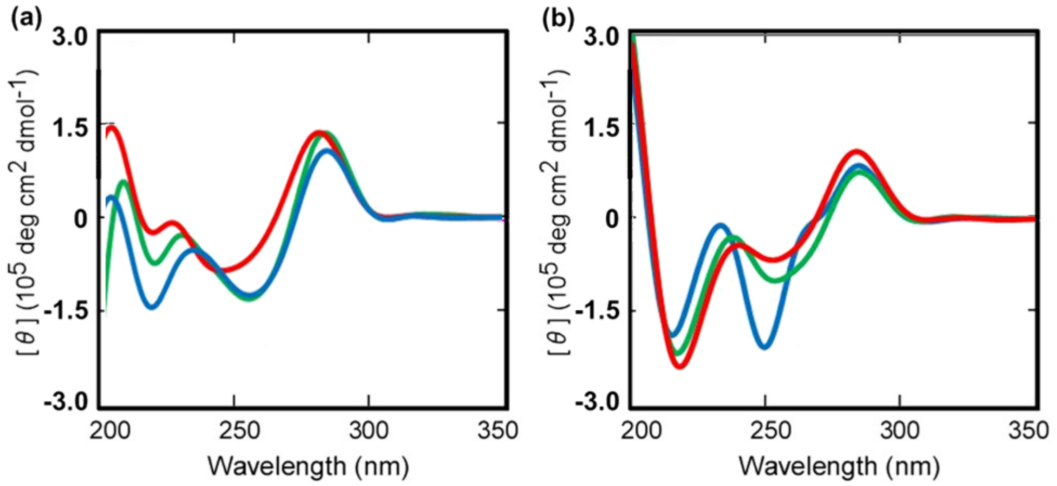

Figure S2. Circular dichroism spectra of 30 μM Ts1 (blue), Ts2 (green) and Ts3 (red) in a buffer containing 50 mM Tris (pH 7.0), 1 mM Na_2EDTA and (a) 4 M NaCl or (b) 4 M choline dhp at 4 °C. The negative peak around 218 nm is typical for a triplex containing Hoogsteen base pairs. This negative peak was very small for Ts3 at pH 7.0 in a NaCl solution, indicating that the Hoogsteen base pairs were destabilized or not formed.

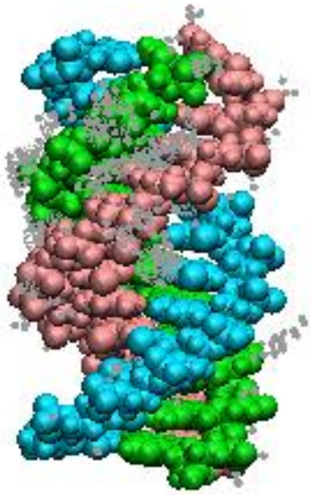

Figure S3. Binding sites of sodium ions (grey dots) around Ts1

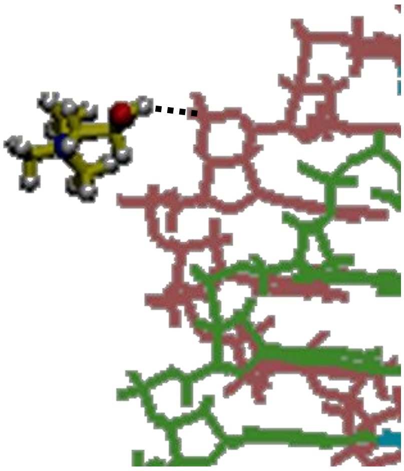

Figure S4. Typical binding mode of a choline ion to the third strand in Ts1. The hydrogen bond is represented by the dashed lines.

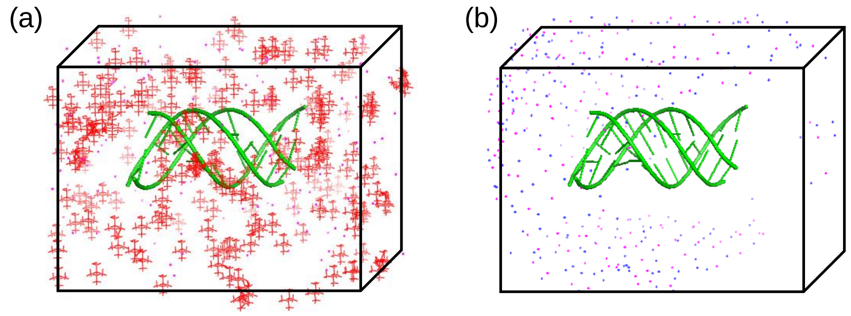

Figure S5. Initial structure of (a) Ts1 containing choline ions and (b) Ts1 containing Na^+ used in our molecular dynamics simulations. DNA chains are shown in green, and choline ions are shown in red. Na^+ and Cl^- are plotted in blue and pink, respectively. Simulation boxes drawn with solid lines were filled with TIP3 water molecules. Water molecules are omitted to clarify the locations of ions.

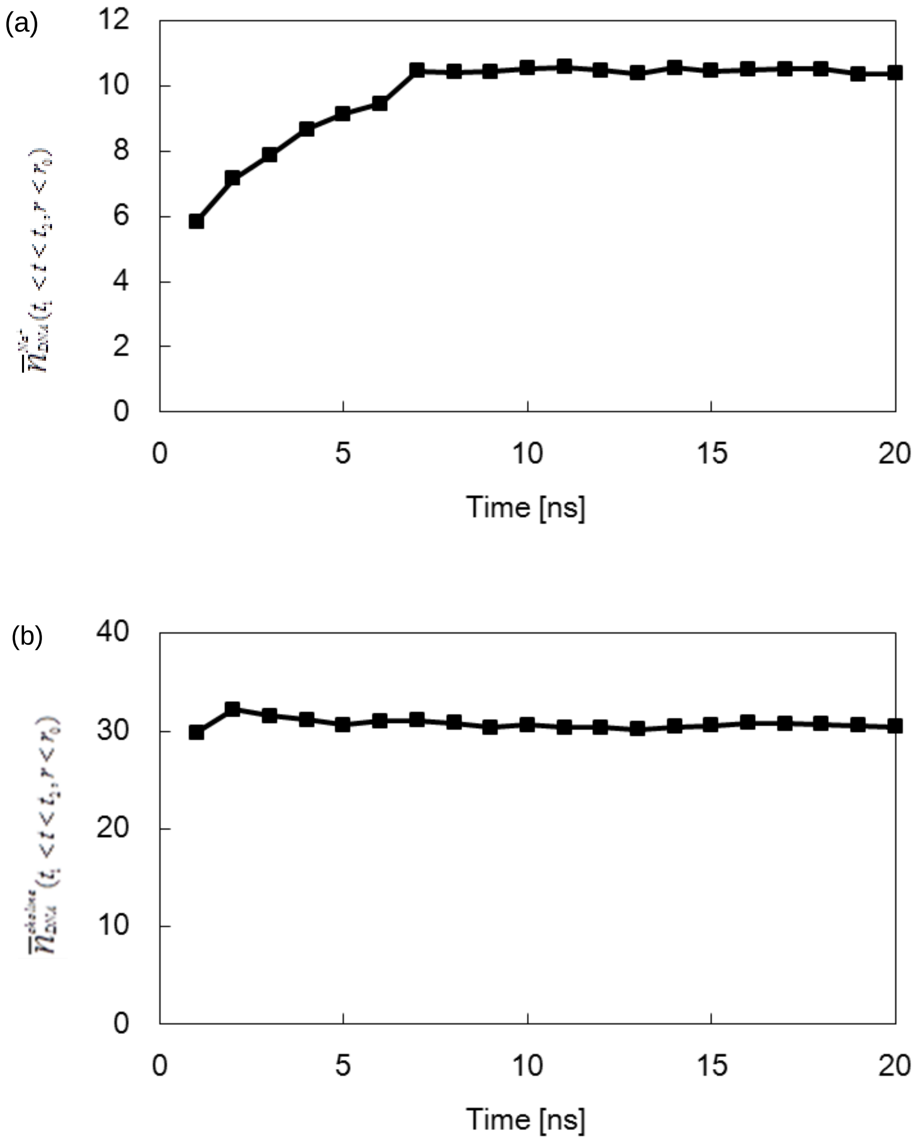

Figure S6. Accumulated averages of (a) Na^+ and (b) choline ions with time within 3.5 Å from DNA atoms. $\overline{n}_{DNA}^{cation}(t_1 < t < t_2, r < r_0)$ is defined in the main text.

(a)

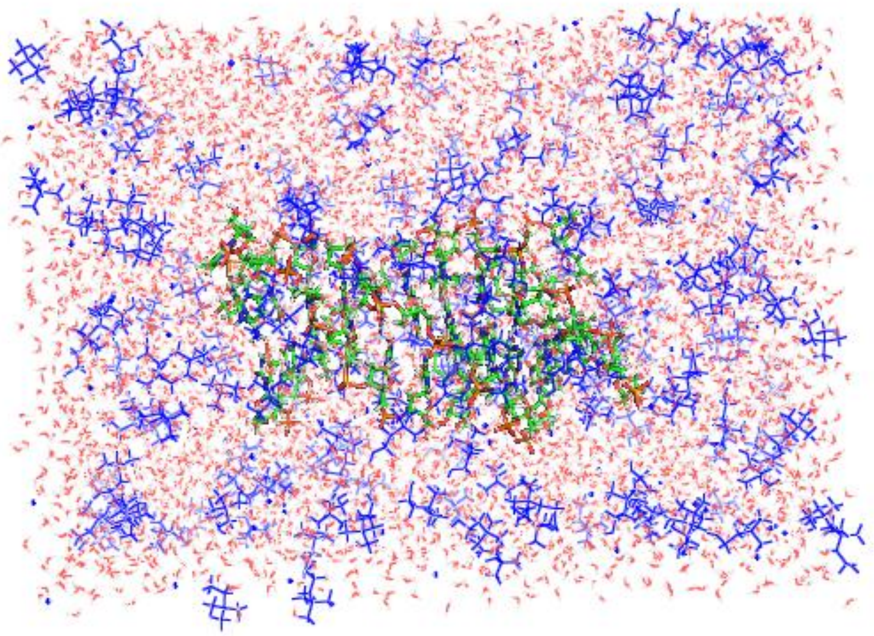

(b)

Name	Number of choline ions	Number of water molecules	Number of atoms
Ts1	33	11381	35867

Figure S5. (a) Initial structure of Ts1 and choline ions used in molecular dynamics simulation. DNA chains are shown in green and choline ions in blue. Oxygen atoms of water molecules are shown in red. Hydrogen atoms are omitted for clarity. (b) Names and properties of the models used in the molecular dynamics simulations.
